# High-Speed Atomic Force Microscopy Reveals the Structural Dynamics of the Amyloid-β and Amylin Aggregation Pathways

**DOI:** 10.3390/ijms21124287

**Published:** 2020-06-16

**Authors:** Takahiro Watanabe-Nakayama, Bikash R. Sahoo, Ayyalusamy Ramamoorthy, Kenjiro Ono

**Affiliations:** 1 WPI Nano Life Science Institute, Kanazawa University, Kakuma-machi, Kanazawa 920-1192, Japan; 2 Biophysics Program, Department of Chemistry, Macromolecular Science and Engineering, and Biomedical Engineering, The University of Michigan, Ann Arbor, MI 48109-1055, USA; bsahoo@umich.edu; 3 Biophysics and Department of Chemistry, University of Michigan, Ann Arbor, MI 48109-1055, USA; ramamoor@umich.edu; 4 Division of Neurology, Department of Internal Medicine, School of Medicine, Showa University, Hatanodai, Shinagawa district, Tokyo 142-8666, Japan; onoken@med.showa-u.ac.jp

**Keywords:** molecular imaging, amyloid β-peptides, islet amyloid polypeptide, high-speed atomic force microscopy

## Abstract

Individual Alzheimer’s disease (AD) patients have been shown to have structurally distinct amyloid-β (Aβ) aggregates, including fibrils, in their brain. These findings suggest the possibility of a relationship between AD progression and Aβ fibril structures. Thus, the characterization of the structural dynamics of Aβ could aid the development of novel therapeutic strategies and diagnosis. Protein structure and dynamics have typically been studied separately. Most of the commonly used biophysical approaches are limited in providing substantial details regarding the combination of both structure and dynamics. On the other hand, high-speed atomic force microscopy (HS-AFM), which simultaneously visualizes an individual protein structure and its dynamics in liquid in real time, can uniquely link the structure and the kinetic details, and it can also unveil novel insights. Although amyloidogenic proteins generate heterogeneously aggregated species, including transient unstable states during the aggregation process, HS-AFM elucidated the structural dynamics of individual aggregates in real time in liquid without purification and isolation. Here, we review and discuss the HS-AFM imaging of amyloid aggregation and strategies to optimize the experiments showing findings from Aβ and amylin, which is associated with type II diabetes, shares some common biological features with Aβ, and is reported to be involved in AD.

## 1. Introduction

The relationship between neurodegenerative disease symptoms and the structural dynamics of associated amyloidogenic proteins has been revealed. For amyloid-β (Aβ), different fibril structures and morphologies have been observed in Alzheimer’s disease (AD) patients [1,2]. Therefore, the Aβ polymorphism was correlated with pathological phenotypes. Similarly, fibril polymorphism and the associated toxicity/infectivity was identified in other amyloidogenic proteins, such as human tau, α-synuclein, and yeast prion-derived Sup35. Tau protein amyloid fibrils demonstrated different isoforms and structures between AD [3], Pick’s disease [4], progressive supranuclear palsy (PSP) [5], corticobasal degeneration (CBD) [5], and chronic traumatic encephalopathy (CTE) in tauopathy [6]. α-synuclein fibrils with different structures exhibited different toxicities [7] and caused different symptoms in α-synucleinopathy [8,9]. One of the best studied prion model proteins, yeast Sup35, formed fibrils with different structures and mechanical stiffness depending on the temperature during aggregation, which was reflected in prion activities, such as fragmentation and propagation [10,11,12]. Elucidating the structural dynamics of the amyloid protein is indispensable to uncovering the mechanism underlying disease onset and the action of drug candidates. Examination of the structure could lead to the diagnosis and treatment methods for the progression of the disease in each individual [13].

Amyloidogenic proteins commonly have intrinsically disordered regions in some or all of their monomeric structure, and they change these to form aggregation core structures along the aggregation pathway. The structural dynamics during the aggregation process depend on the surrounding physicochemical conditions in vivo, including the free-in-solution or membrane-bound forms, pH, and electrolytes. Aβ40, a 40 residue Aβ variant cleaved from the amyloid precursor protein (APP), is unstructured [14,15] and can also form a lowly populated 3_10_ helical structure in solution [16]; disordered oligomers were also reported [17]. This variant formed disordered helical structures upon interaction with the lipid membrane [18], and could disrupt the membrane structure via a two-step mechanism consisting of fiber-independent pore formation and fiber-dependent ‘detergent-like’ membrane fragmentation [19]. Aβ42—a 42 amino acid chain of Aβ variants—showed different structural dynamics from Aβ40 through the interaction with a membrane. Aβ42 oligomerization was accelerated by the lipid membrane [20]. Aβ42 oligomers assembled into the pore-forming oligomers with three distinct pore sizes that functioned as ion channels, while Aβ40 did not form such pores [21]. Upon interaction with a reconstituted membrane, Aβ42 assembled into a β-barrel structure, while Aβ40 formed fibrils [22]. Tau [23], α-synuclein, and other amyloidogenic proteins, including amylin, also formed ion-channel oligomers in the membrane [24]. Cytosolic acidification caused by oxidative stress promoted AD [25] and Parkinson’s disease (PD) [26]. A change in pH altered the structural dynamics and aggregation pathways of Aβ [27,28,29,30], tau [31], α-synuclein [32,33,34,35], and amylin [36]. Some amyloid oligomeric conformers were more toxic than amyloid fibrils [37,38,39]. Different oligomers showed different structures and toxicities [40,41]. Thus, amyloid fibrils with different structures may reflect variable toxicity, aggregation pathways, and surrounding microenvironments in different patients and symptoms [1,42].

To date, investigations of the structural dynamics of amyloidogenic protein aggregation examined the structure and dynamics separately. X-ray crystallography, nuclear magnetic resonance (NMR), and cryo-electron microscopy (cryo-EM) revealed the spatial coordinates of constituent atoms in the protein structure. X-ray crystallography demonstrated the fibril structures of amyloidogenic protein fragments [43,44] and oligomers of Aβ peptides [45,46,47,48]. NMR studies reported the high-resolution structural details of membrane-bound amyloid oligomers [41,49,50]. Solution- and solid-state NMR was used to investigate the monomer and transient intermediate structures in Aβ assemblies [16,51,52,53,54,55,56,57,58]. In addition, solid-state NMR studies revealed the in-register parallel β-sheets in Aβ fibril structures [1,42,59,60,61,62,63,64]. Cryo-EM studies discovered the Aβ42 fibril structure [65] and individual Aβ40 strain structures from AD patients [2]. However, the structural images were static and average across the applied protein samples. Thus, those methods require structural homogeneity in the analyzed protein samples.

Single molecule observation using fluorescence dyes under optical microscopy was used to visualize the structural dynamics of amyloidogenic protein aggregations. Thioflavin T (ThT), specific for the cross-β structure, [66,67] was used to visualize the fibril elongation of Aβ40 [68], α-synuclein [69], and prion protein (PrP) [70]. Other fluorophores co-assembled or cross-linked to proteins were used to visualize the aggregation dynamics of α-synuclein [71,72,73,74,75,76] and the fibril growth of Aβ [77] and Sup35 [78]. However, the captured images did not show the protein structure but the spatial distribution of fluorescence spots.

Atomic force microscopy (AFM) visualizes individual molecules at the nanometer spatial resolution in solution, although the temporal resolution is low in conventional AFM [79,80,81,82,83,84]. AFM was used to capture structural images and measure the nanomechanical properties of individual amyloid aggregates [85,86,87] in the ongoing heterologous aggregation processes of Aβ [20,88,89,90,91,92,93,94,95,96,97,98], synuclein [90,99,100,101,102,103,104,105,106], and amylin [107,108]. High-speed AFM (HS-AFM) enabled the kinetic measurement of the structural dynamics of biological molecular processes [79,80,81,82,83,84] including amyloid aggregation [93,108,109,110,111,112,113,114,115,116]. Here, we show that HS-AFM links structural and dynamics studies, reviewing recent HS-AFM studies and including our findings for Aβ42 [93] and amylin [116], which is associated with not only type II diabetes but also AD [117,118,119,120,121,122,123].

## 2. HS-AFM Observation of Aβ42 Fibril Growth

### 2.1. HS-AFM Observation of Self-Replicative Aβ42 Fibril Growth

Aβ fibril formation has been thought to be similar to the self-replication mechanism for prion proteins. In this mechanism, the soluble conformer assembles and changes its structure to an abnormal form, and some of the abnormal conformers grow into fibrous structures with the incorporation of the soluble conformers [60]. The time-lapse AFM with a lower scanning rate observed Aβ40 protofibrils and mature fibrils growing in a self-replicative manner [89,107]. To assess Aβ42, characterized by faster aggregation, we used HS-AFM.

**Figure 1:**
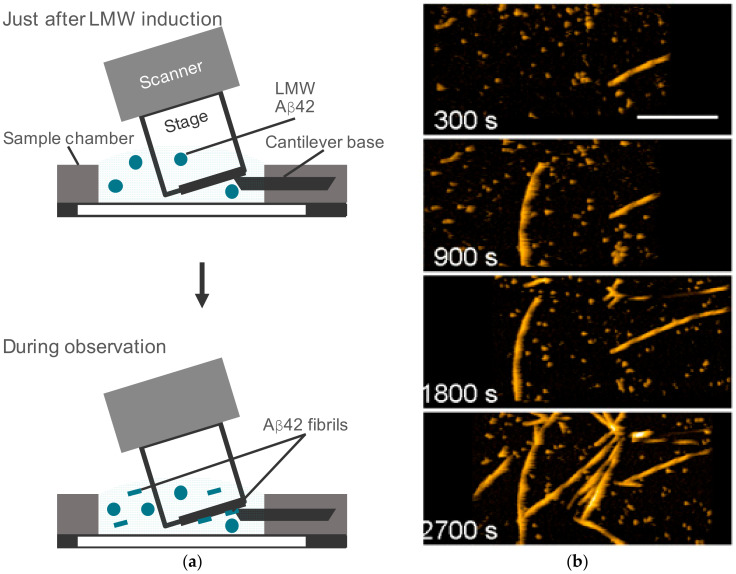
High-speed atomic force microscopy (HS-AFM) observation of amyloid-β (Aβ)42 fibril elongation. (**a**) Schematic view of HS-AFM observation of low molecular weight (LMW) Aβ42 incubation. LMW Aβ42 in 10 mM sodium phosphate, at pH 7.4, was introduced with 0.1 M NaCl for aggregation acceleration. Some aggregates in the solution bound to the mica surface and the fibrous aggregates in them were elongated by the incorporation of the LMW Aβ42 in free solution. (**b**) Representative HS-AFM images of LMW Aβ42 incubation at the indicated time after addition of 0.1 M NaCl. The scale bar is 300 nm. Reproduced from [93].

The sample preparation procedure and the imaging conditions were critical for the HS-AFM observation, as described in Section 8.1. We prepared low molecular weight (LMW) and high molecular weight (HMW) Aβ42 fractions in 10 mM sodium phosphate, with pH 7.4 [93,124]. Aβ42 fibril formation and elongation was observed in LMW Aβ42 within approximately 1 h after the addition of 0.1 M NaCl [93] (Figure 1). The LMW Aβ42 was introduced to the HS-AFM sample chamber (Figure 1a). Sodium chloride was added just before or after the peptide introduction; this was immediately followed by HS-AFM observation. Species in solution were adsorbed to the surface and became detectable by HS-AFM (Figure 1a,b) [93]. The bound aggregates interacted with species in the solution and were able to elongate as shown in Figure 1b.

**Figure 2:**
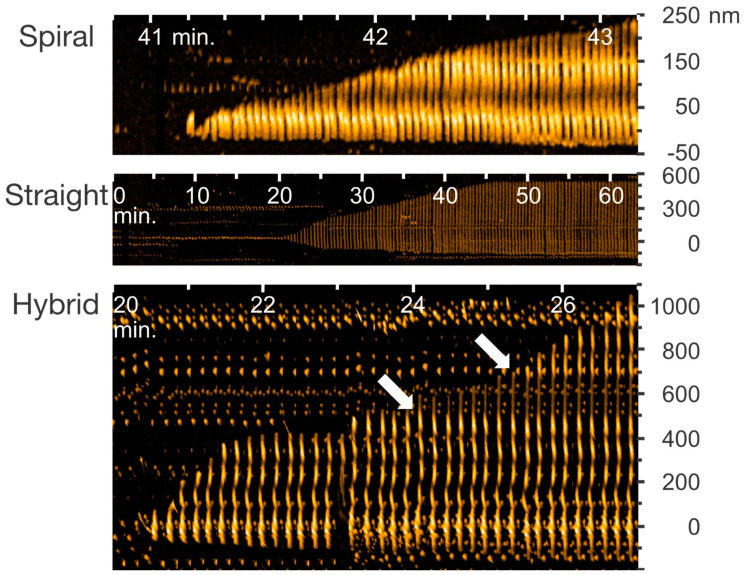
Kymographs of spiral, straight, and hybrid types of Aβ42 fibrils from HS-AFM images of the indicated time ranges after addition of 0.1 M NaCl. Arrows indicate the positions at which the growth mode switched from the spiral to the straight at 24 min and from the straight to the spiral at around 25 min. Reproduced from [93].

The HS-AFM images clearly distinguished the three structurally distinct types of fibrils in this condition: (1) the spiral fibrils with a ≈100 nm periodicity in height, (2) the straight fibrils without any structural periodicity, and (3) the hybrid fibrils in which the spiral and the straight parts were mixed (see Figure 2) [93]. This result indicates that the manner of Aβ42 fibril growth followed the prion-like self-replication in the spiral and the straight parts [93]. In addition, HS-AFM also uncovered the structural switch in the hybrid fibril elongation and the presence of spherical oligomers in the sample mixture [93]. A recent solution NMR study identified fibril elongation and the formation of a heterogeneous mixture (fibrils and oligomers) when Aβ monomers were added to sonicated preformed seeds in real time [125]. However, a solid-state NMR investigation is needed to obtain additional high-resolution structural information; whereas, HS-AFM experiments eased the retrieval of the structural switch and fibril polymorphism in real time.

**Figure 3:**
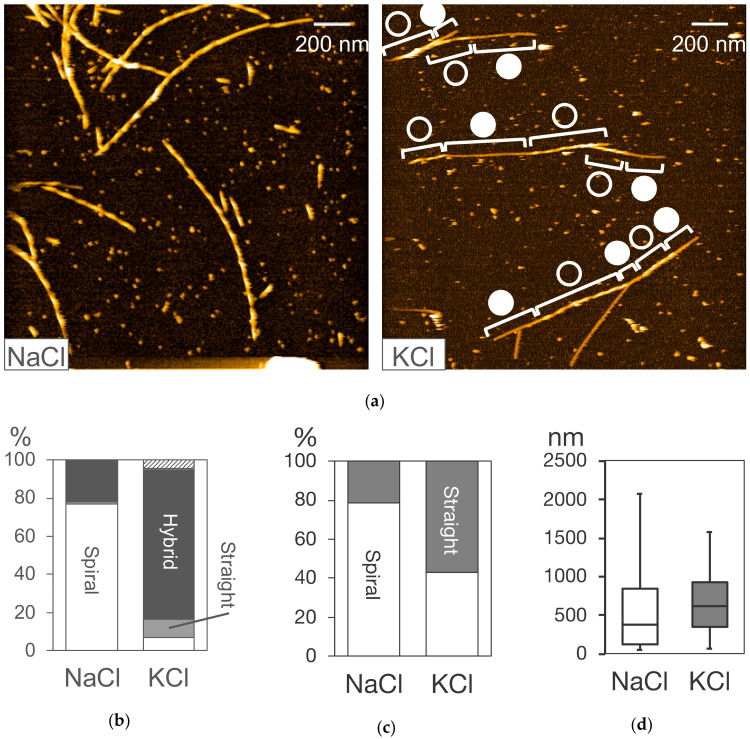
HS-AFM observation of fibril elongation from LMW Aβ42 incubation on mica surface in 10 mM sodium phosphate, pH 7.4 titrated with 0.1 M NaCl, or KCl. (**a**) HS-AFM images of Aβ 42 fibrils approximately 1 h after the addition of NaCl or KCl as indicated. The spiral and straight parts in the hybrid-type fibrils are indicated by open and closed circles. (**b**–**d**) Distributions of the fibril type (**b**), the growth mode (**c**), and the fibril length (**d**) under NaCl and KCl conditions. The top hatched portion for KCl in (**b**) indicates the fibrils whose structure was not determined due to their length being shorter than the spiral pitch. Reproduced from [93] except for the image in NaCl shown in (**a**).

The molecular process of Aβ aggregation was characterized by HS-AFM observation under various physicochemical conditions. The structural switch of the growth mode was also characterized to be an inherent process in the Aβ42 self-replication reaction. To characterize the structural switch, Aβ42 fibril formation and elongation was observed after the addition of 0.1 M sodium chloride or potassium chloride [93]. The interaction between the HS-AFM stage surface and the observed Aβ42 molecules was different in the presence of sodium and potassium ions, as described in Section 8.3. As shown in Figure 3a, the fibril type distribution was different under the sodium and potassium conditions [93]. The spiral and the hybrid types were dominant in the sodium and potassium ion buffers, respectively, with no significant difference in the fibril length between the two conditions (Figure 3a,b,d) [93].

The appearance frequency of the spiral and the straight growth modes decreased and increased in the potassium buffer, respectively, when compared with the sodium buffer (Figure 3c) [93]. The lengths of the spiral and straight sections in the potassium condition were shorter and longer than in the sodium condition, respectively [93]. These results indicated that the structural switch process could be modulated by changes in the physicochemical conditions, and that the sodium buffer constrained fibril growth to the spiral mode, while the potassium buffer decreased the activation energy and the free energy difference between the spiral and the straight states of the structure switch process [93]. These findings revise the conventional model of the self-templating replication of amyloid fibrils, suggesting that the fibril structure strain can be altered by the external physicochemical environment even after fibril seed formation (nucleation) [93].

### 2.2. Kinetic Analysis of Aβ42 Fibril Growth

**Figure 4:**
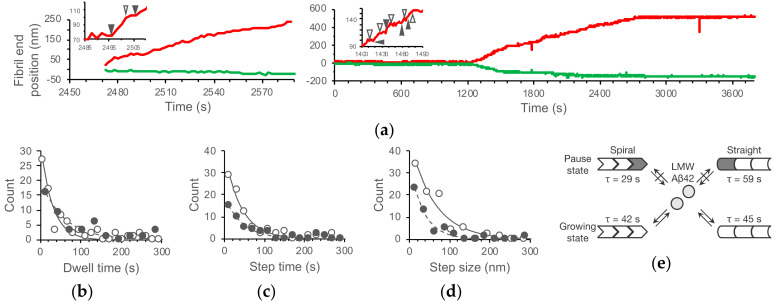
Stepwise growth of Aβ42 fibrils. (**a**) Time courses of the fast (red) and the slow (green) ends of the spiral and straight fibrils in Figure 2. The insets are the enlarged time courses of the fast ends. The open and closed triangles indicate the start and end of pause states (dwell time). (**b**–**d**) Distribution of the dwell time (**b**), time for step (**c**), and step size (**d**) with single exponential fits giving the mean life times of the pause and growing states in (**e**), and mean step sizes, for the spiral (open circles with solid lines) and straight (closed circles with dashed lines) type fibrils from the LMW Aβ42 incubation. (**e**) Mechanistic model of the stepwise growth of the spiral and straight Aβ42 fibrils with the kinetic parameters from (**b**) and (**c**). Reproduced from [93].

The high spatiotemporal resolution of HS-AFM enables kinetic analysis of fibril elongation according to the fibril structure. Aβ42 fibrils, especially at the fast ends, repeated the pause (dwell) time and the growth phase during their elongation in both the spiral and the straight growth modes (Figure 4a) [93]. The dwell time, step time, and step size showed the single exponential distributions (Figure 4b), suggesting that the transition between the dwell phase and growth phase proceeds with first-order kinetics [93]. The kinetic parameters for this transition were different between the spiral and straight growth modes (Figure 4c) [93]. The step sizes (62 nm and 36 nm of mean values for the spiral and the straight growth modes, respectively) were much longer than the width of the single β-strand (0.47 nm) in amyloid fibrils, which suggests that a number of peptides were taken into the fibrils during the growth phase, and that the transition between the dwell and growth phases did not correspond to the incorporation or dissociation of peptides (peptide concentration independence of the dwell phase needs to be examined) (Figure 4d).

The similar stepwise growth and its kinetics were characterized in Aβ25–35 fibril growth by AFM [126]. The transitions between the pause and growing phases were also identified as first-order kinetic processes with the structure conversion at the fibril ends [126]. The fibril ends can neither incorporate nor release peptides at the pause phase (the blocked state), while they consecutively grow with 7 nm or its integer multiples during the growing phase [126]. The differences in the kinetic parameters between the spiral and the straight growth modes of Aβ42 indicate that the energy landscape for the transition between the dwell and growth modes was different between the two types of fibrils. The fibril end structure at the dwell phase may also be different between the spiral and straight fibrils (Figure 4e).

## 3. Mechanical Force Modulates the Transition from Oligomeric to Filamentous States of Aβ

HS-AFM can be used to investigate the force-dependent molecular processes. The physical environment is also one of essential factors that determine biochemical responses [127]. In the amyloid research field, the application of external mechanical forces, such as shaking, agitation, and sheer stress, has been used to accelerate aggregation and fibrillation [128,129,130,131,132]. A recent study by Wang et al. showed the effects of mechanical sample rotation in the commonly used magic angle spinning NMR experiments to study amyloid-β aggregation and the effect of (−)-epigallocatechin gallate (EGCG), which is a small molecule polyphenolic compound found in green tea extract [133]. However, the underlying mechanism of the physical effects has remained unclear. Although the exerted force to the sample by the AFM tip is usually kept as small as possible (see Section 8.4), methods for controlling and actively utilizing the applied force to characterize the molecular processes have been developed [134,135].

**Figure 5:**
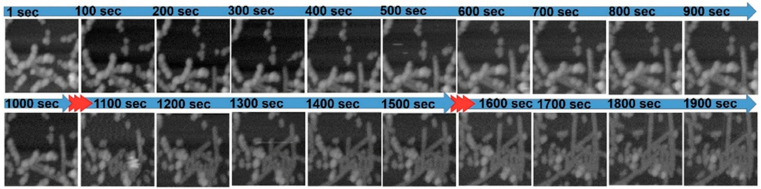
HS-AFM imaging of tip-induced Aβ42 filament growth. The HS-AFM tapping amplitude was transiently increased at the time indicated by the red arrows. Reprint with permission [112]; Copyright (2019), John Wiley and Sons.

Using the external force applied by HS-AFM, Tashiro et al. found the linearly organized globular Aβ42 oligomers that showed the force-dependent thin filament extrusion [112]. They first observed the aggregate species in 1-day Aβ42 incubation bound to the stage surface, and then temporarily increased the tapping force of the cantilever tip, which is immediately followed by the observation of the same area with the original tapping force [112]. As shown in Figure 5, the breakage of the connected oligomers induced the extension of the thin straight filaments [112], which suggests that the stability of the oligomers may suppress their structural conversion into the filaments and that its removal may be promoted by the applied force. This result provides insights into the importance of the mechanistic stability of amyloid oligomers.

## 4. HS-AFM Observation of Aβ42 Aggregates on the Off-Pathway

### 4.1. HS-AFM Observation of Dissociating Aβ42 Aggregates

**Figure 6:**
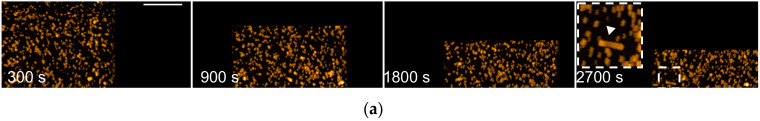

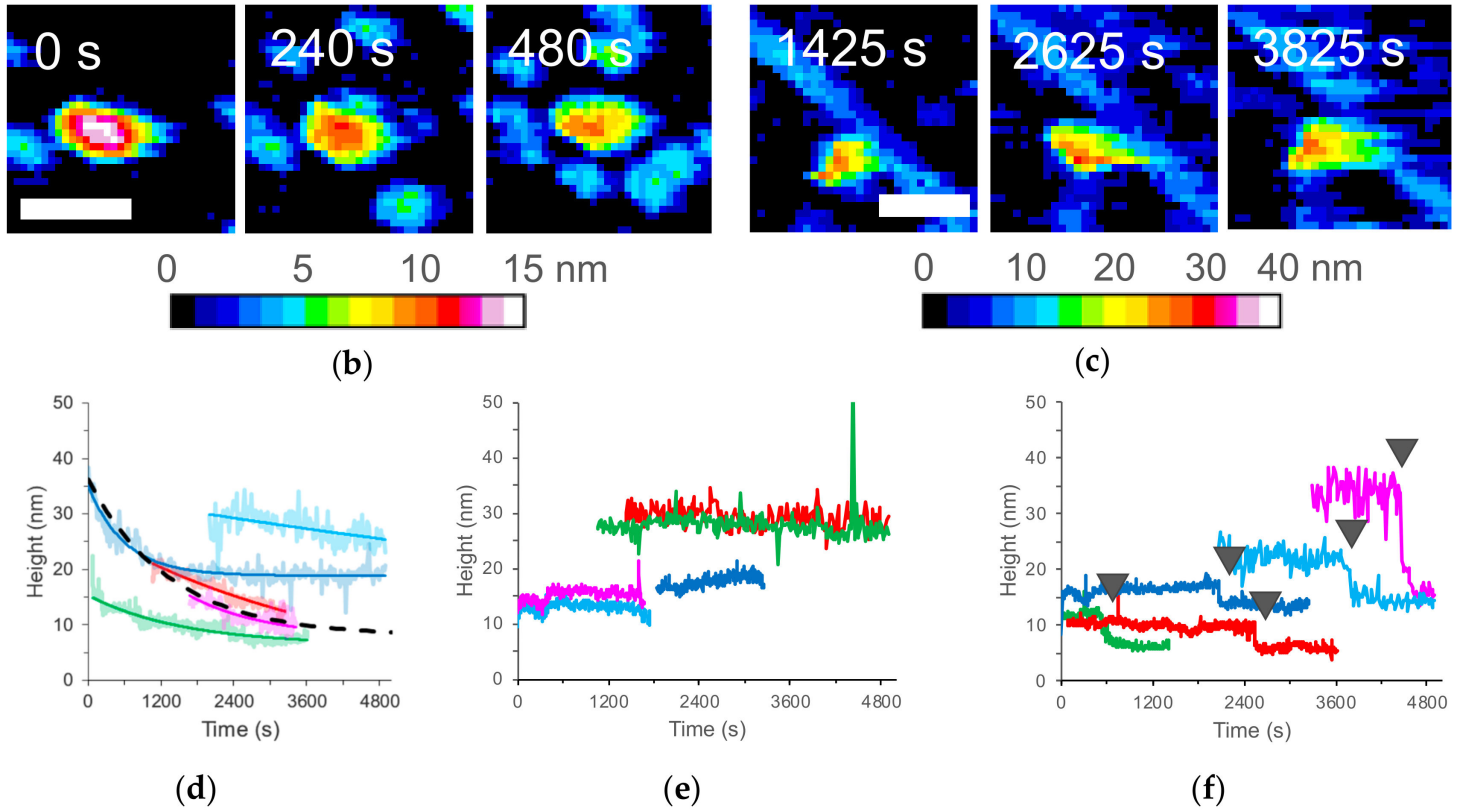
HS-AFM observation of non-fibrous aggregates in high molecular weight (HMW) Aβ42 incubation. (**a**) HS-AFM images of HMW Aβ42 incubation at the indicated time after the addition of 0.1 M NaCl. A closed triangle in the highlighted dashed box indicates a representative short fibril in HMW incubation. Bar, 300 nm. (**b**,**c**) HS-AFM image of two types of spherical aggregates in HMW incubation: type I gradually decreased in height (**b**); type II maintained its height (**c**). Bars, 50 nm. (**d**–**f**) Five representative time courses of height of type I (**d**), type II (**e**), and type III (**f**) aggregates in HMW incubation after an addition of 0.1 M NaCl. Each of the trajectories for type I is shown with the fitting curve of Equation (2). The dashed line corresponds to Equation (2), with the median values obtained from the best-fit values for individual type I aggregates. The type III aggregates show the sudden decrease in their heights at the time indicated by closed triangles. Different colors correspond to different single aggregates. Reproduced from [93].

HS-AFM visualizes the structural dynamics of non-fibrous Aβ42 aggregates. The HMW fraction, which was obtained by gel filtration, mainly contained globular aggregates [93]. They were classified into three different types of structural dynamics after the addition of 0.1 M sodium chloride: (1) gradually dissociating aggregates, (2) aggregates with unchanging sizes, and (3) stepwise dissociating aggregates (Figure 6a–f) [93]. The type I aggregates exponentially decreased in size (measured as the height from the HS-AFM stage) and then reached certain non-zero sizes, which suggests that the type I aggregates were composed of at least two parts with different dissociation constants (Figure 6d) [93]. The time course of the type I size, h(t), can be expressed as Equation (1):

h(t) = h_a0_·exp(-k_a0_·t) + h_b0_·exp(-k_b0_·t) (1)

where h_a0_ and k_a0_ are the initial height and dissociation constant of part a, and h_b0_ and k_b0_ are the initial height and dissociation constant of part b. When the dissociation rate is extremely low (k_b0_ ≒ 0), Equation (1) can be rewritten as Equation (2):

h(t) = h_a0_·exp(-k_a0_·t) + h_b0_. (2)

Equation (2) fitted the time courses of the type I aggregates well (Figure 6d) [93]. These analyses indicated that the type I aggregates formed during the sample preparation in 10 mM sodium phosphate, pH 7.4, and were then dissociated by the equilibrium shift after the addition of 0.1 M sodium chloride. For the relationship between the type I, II, and III aggregates, there were at least two possibilities: (1) they were distinct assemblies or (2) type II and III correspond to the slow dissociating part (the part b) of the type I aggregates [93]. The fast dissociating parts were completely dissociated before the aggregates appeared in the observation area, which may correspond to the type II aggregates (Figure 6b,d) [93]. We should also note that the tapping AFM probe may accidentally break the type II aggregates, resulting in a stepwise decrease in the aggregate size and thus generate type III aggregates (Figure 6f) [93].

### 4.2. Characterization of Aggregation Pathway by Statistical Analysis

**Figure 7:**
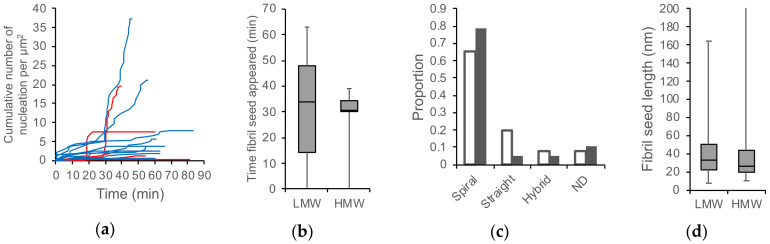
Timing of the fibril appearance in the incubation of LMW and HWW Aβ42. (**a**) Time courses of cumulative number of fibrils in the observation area from LMW (blue lines) and HMW (red lines) Aβ42 incubation. Different lines correspond to different experiments. (**b**–**d**) The distribution of times at which individual fibril seeds appeared (**b**), the fibril type (**c**), and the length of fibril seeds when they first appeared in the observation area (**d**). The open and closed bars in (**c**) correspond to LMW and HMW incubation, respectively. Reproduced from [93].

HS-AFM analysis of the frequency of appearance of specific amyloid aggregates identified the position of individual aggregates during the molecular process. Figure 7a shows the time course of the cumulative number of fibrils appearing in the observation area after the addition of 0.1 M sodium chloride to the LMW or the HMW Aβ42 fractions [93]. The graph shows an upward trend in which fibrils appeared and accumulated. Although the LMW appeared throughout the time period, the HMW began to increase in the number of fibrils 20 to 30 min after the incubation. This tendency can be confirmed remarkably on the distribution of the time when the fibrils appeared, and there was a significant difference in the variance of the two distributions (Figure 7b) [93]. The bulk assay with gel filtration also indicated that the HMW incubation temporarily produced the LMW fraction [93]. There was no difference in the distribution of the fibril types between the incubation of the LMW and the HMW Aβ42 (Figure 7c) [93]. No significant difference was observed in the size of the fibril seeds at the time of appearance (Figure 7d) [93]. These results suggest that the on-pathway for fibril formation was common to both the LMW and HMW Aβ42 incubations, and that the HMW took time to form fibril nuclei [93]. As shown in Section 4.1, the HMW contained aggregates that dissociated according to the equilibrium shift after the addition of 0.1 M sodium chloride. Therefore, the HMW was located in the off-pathway and dissociated to generate the LMW; then, the fibrils were formed along the on-pathway [93]. This HS-AFM observation correlated to a recent kinetic study reported by Knowles et al., which calculated a fast Aβ42 oligomer formation (approximately 8 × 10^−7^ S^−1^) and slow oligomer dissociation (approximately 9 × 10^−5^ S^−1^) [136]. Thus, the delay in HMW dissociation to form a seed competent LMW is connected to the delay in fibril elongation.

## 5. HS-AFM Observation of Early Aggregation Stages of Aβ

Although highly challenging, there is considerable interest in probing the early events of amyloid aggregation to trace the formation of toxic oligomeric intermediates. While several biophysical approaches, including a combination of NMR techniques, have been employed to monitor these events at high resolution [48], they are limited by many factors, including the sample size, sensitivity, and timescale for measurements. On the other hand, HF-AFM is well suited for this purpose and can complement well with other studies by providing high-throughput measurements in real time.

**Figure 8:**
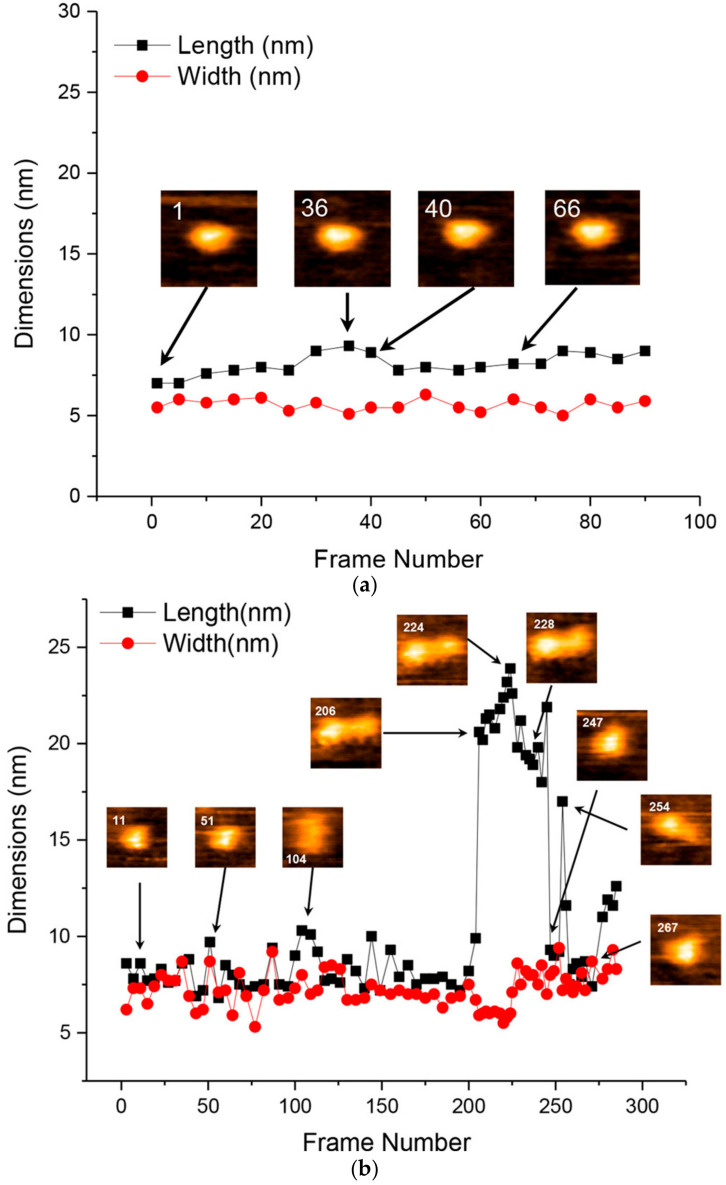

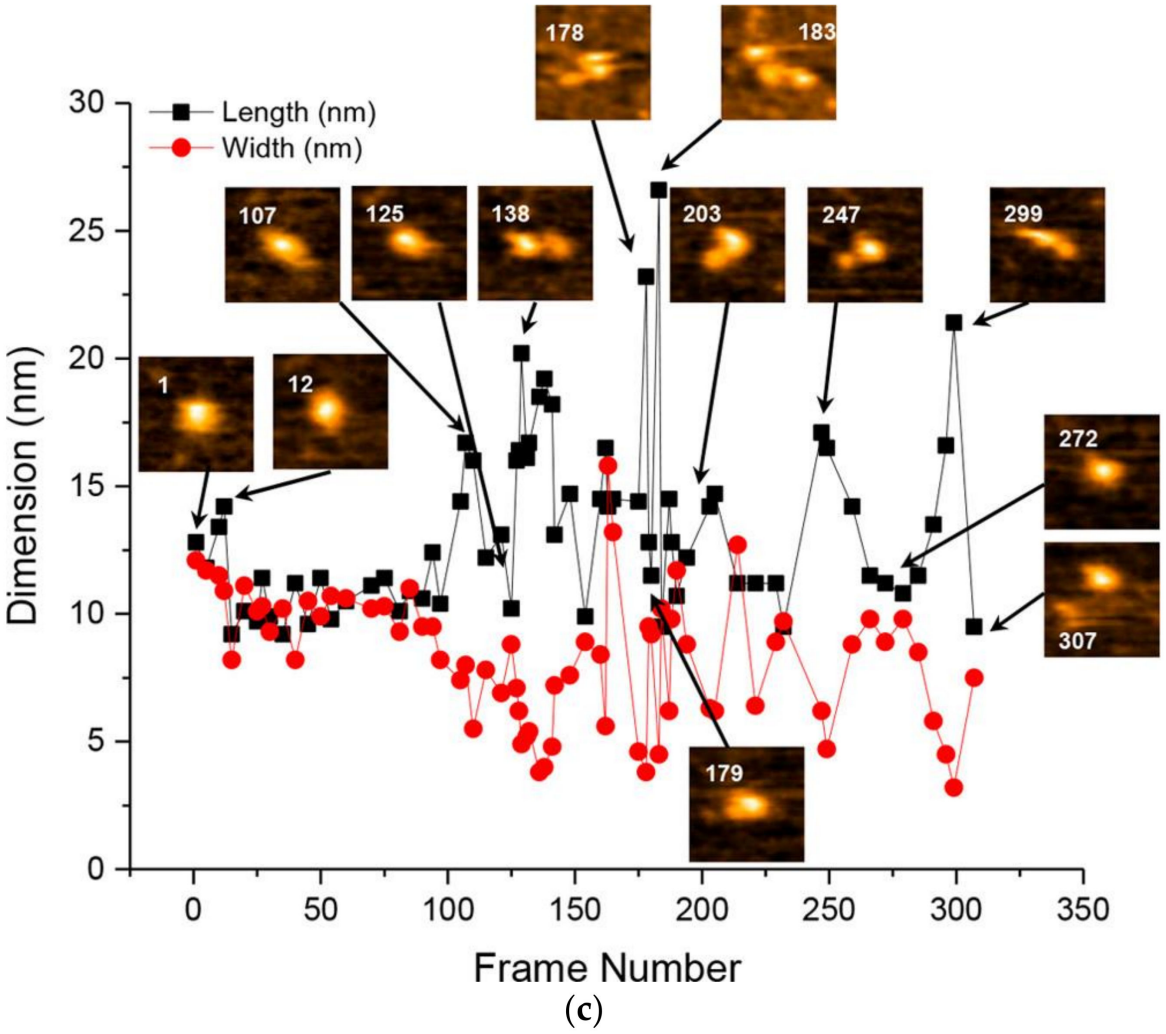
HS-AFM images of Aβ42 oligomers. Time courses of width and length obtained from HS-AFM images at the indicated times for trimers (**a**), pentamers (**b**), and heptamers (**c**). Reprinted with permission from [109]. Copyright (2017) American Chemical Society.

Banerjee et al. characterized the structural features of low-order Aβ42 aggregates, measuring the spatial size of monomers through decamers and observing intramolecular structural dynamics of trimers, pentamers, and heptamers [109]. They prepared stable oligomers up to decamers by photochemical cross-linking and measured the sizes [109]. They found that the oligomeric order/size (volume) relationship showed two different proportionalities with the boundary at the tetramer, suggesting that at least two types of monomer packing patterns exist in their assembly [109]. Using HS-AFM, they observed intramolecular structural dynamics of the representative oligomers (trimer, pentamer, and heptamer) at subsecond temporal resolution [109]. The trimers sustained single blobs with almost constant width and length, while the pentamers and heptamers showed transition between a single compact globular shape and the extended multi-lobe structure (Figure 8). The pentamer had the two blobs with different sizes to each other in the extended state (Figure 8b). The heptamer can be extended to three lobes with different size, in addition to the double-lobe state (Figure 8c). These results suggest that Aβ42 oligomers are the lobe-linked structures, in which each lobe is composed of two or three peptides and that each oligomeric state can transiently extend and shrink between a compact globular shape and the multi-lobe structure [109].

**Figure 9:**
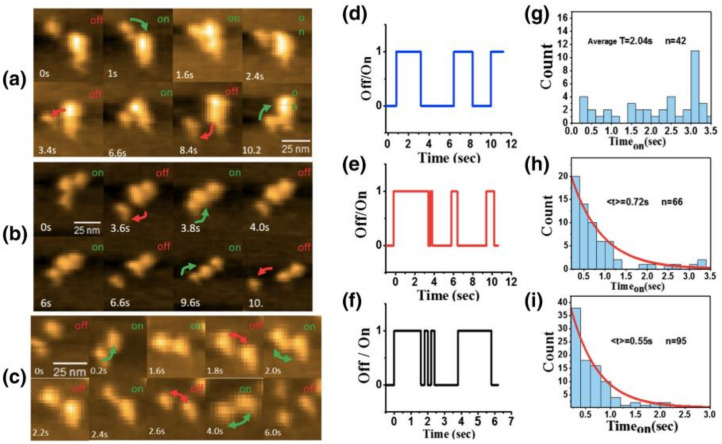
HS-AFM imaging and kinetic analyses of interaction between Aβ oligomers. Successive HS-AFM images (**a**–**c**), representative time courses of binding/dissociation (**d**–**f**), and distributions of the bound state (**g**–**i**) for the interaction of Aβ_15–20 nm_–Aβ_Agg_ (**a**,**d**,**g**), Aβ_15–20 nm_–Aβ_36 nm_ (**b**,**e**, and **h**), and Aβ_15–20 nm_–Aβ_15–20 nm_. Reprint with permission [113]: Copyright (2019), Elsevier.

Feng et al. identified that Aβ42 at the early aggregation stage can be classified into four structural types and characterized the kinetic interactions between those aggregates using HS-AFM [113]. They observed Aβ42 aggregates shortly after the dissolution of 1,1,1,3,3,3-hexafluoro-2-propanol (HFIP)-treated Aβ42 in phosphate-buffered saline. Their statistical analyses based on the three dimensional size measurements indicated four classes of Aβ42 aggregates: Aβ_15-20 nm_ with 15–20 nm (length and width) and height of 2.8 nm; Aβ_36 nm_ of a bilobed structure with 34 nm length, 17 nm width, and 2.8 nm height; and Aβ_Agg_ with 34–36 nm (length and width) and height of 9.2 nm; disordered chain-like structure [113]. They also observed binding/dissociating interactions between the different type aggregates. The on/off interactions of Aβ_15–20 nm_–Aβ_36 nm_ and Aβ_15–20 nm_–Aβ_15–20 nm_ were respectively characterized to be a single step process [113] (Figure 9b,c,e,f,h,i). The Aβ_15–20 nm_–Aβ_36 nm_ binding showed slightly higher affinity compared with the Aβ_15–20 nm_–Aβ_15–20 nm_ interactions [113] (Figure 9h,i). In contrast, the binding time for Aβ_15–20 nm_–Aβ_Agg_ was distributed randomly (Figure 9a,d,g), which suggests that the Aβ_15–20 nm_–Aβ_Agg_ interaction was not a single step but accepted various binding patterns [113]. Inevitably, this “permissive” interaction may produce various types of oligomers with different toxicities [113].

## 6. HS-AFM Observation of Interaction between Amyloidogenic Proteins and Other Chemical Compounds

HS-AFM can also identify the interactions between amyloidogenic proteins and other chemical compounds, such as lipids and the potential anti-amyloidogenic inhibitor target(s) in the amyloid aggregation pathway by comparing observations in the presence and absence of the compounds.

### 6.1. Interaction between a Toxic Aβ Oligomer and Lipid Bilayer

Membrane–amyloidogenic protein interactions have been thought to lead specific aggregate conformers and play an important role in neurotoxicity as described in the Introduction. Aβ aggregation on a membrane has been known to depend on the physicochemical properties of the membrane (lipid composition, gel/liquid phases, phase separation, charge on head group, and oxidation level), which influences the toxicity of Aβ [137,138,139,140,141,142,143]. HS-AFM can visualize the effect of the lipid–amyloid interaction on the lipid membrane and characterize the interaction in real time.

**Figure 10:**
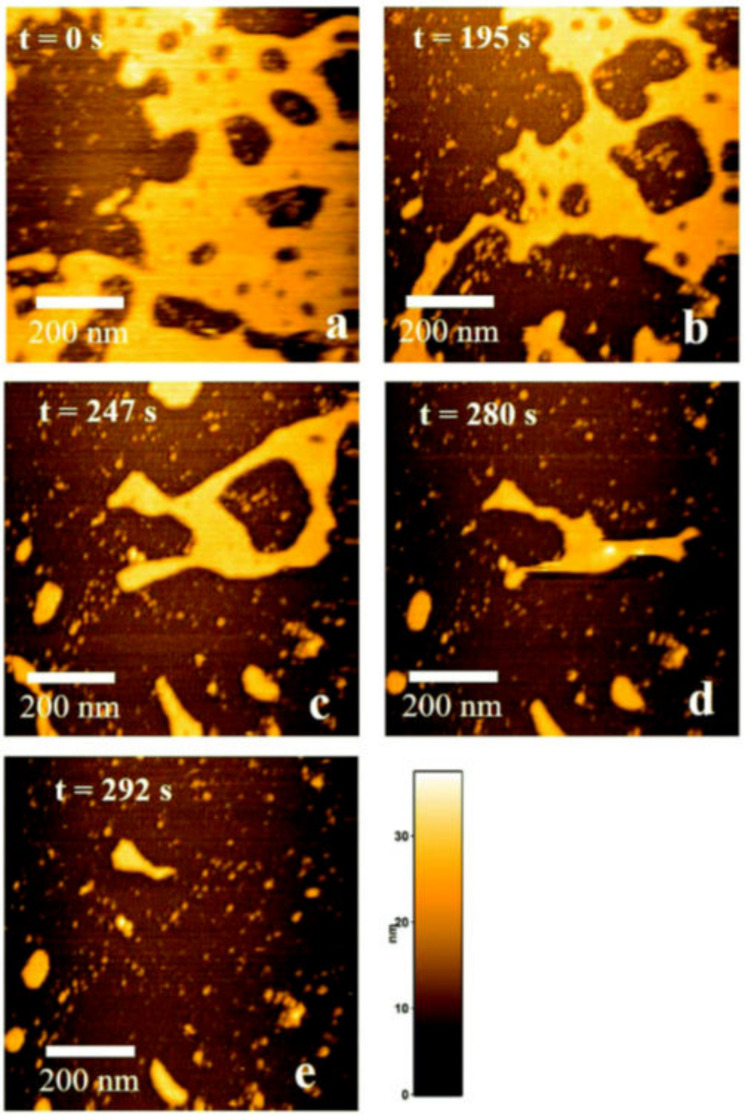
HS-AFM images of sphingomyelin (SM)/1-palmitoyl-2-oleoylphosphatidylcholine (POPC)/cholesterol (Chol)/GM1-ganglioside (GM1) membrane disruption after the addition of Aβ42 oG37C oligomers (**a**–**e**). The membrane with holes was removed from its edge. Reproduced from [111] with permission from The Royal Society of Chemistry.

Ewald et al. demonstrated the importance of the lipid composition on the interaction with a toxic Aβ oligomer, showing HS-AFM movies of lipid composition-dependent membrane disruption [111]. They prepared the toxic Aβ42 oG37C oligomers in which the 37th residue was changed from glycine to cysteine and added them to the planar lipid bilayer on the stage, changing the lipid composition in the mixture of sphingomyelin (SM)/1-palmitoyl-2-oleoylphosphatidylcholine (POPC)/cholesterol (Chol)/GM1-ganglioside (GM1). They visualized the GM1-dependent oligomer anchoring and the requirement of GM1/Chol coexistence at an appropriate ratio for membrane solubilization (Figure 10) [111].

### 6.2. Aggregation Inhibition by Natural Phenolic Compounds

**Figure 11:**
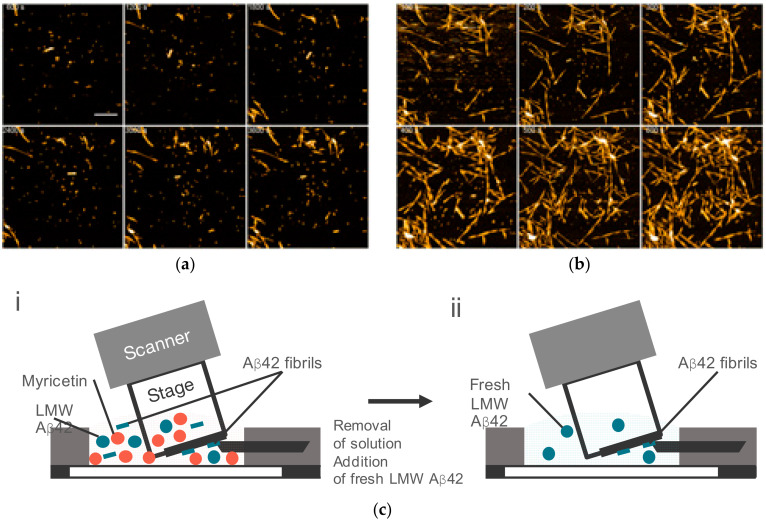
HS-AFM observation of Aβ42 fibril growth in the presence/absence of myricetin. (**a**) HS-AFM images of LMW Aβ42 incubation with fibril seeds and myricetin at the indicated time after the addition of 0.1 M NaCl. Scale bar, 300 nm. (**b**) HS-AFM images of the same observation area after the replacement of the solution to fresh LMW Aβ42. (**c**) Schematic views of HS-AFM observation condition (i) and (ii) correspond to (**a**) and (**b**) Reproduced from [93].

Some natural phenolic compounds in foods have been known to inhibit amyloid aggregation [144,145,146,147,148,149,150,151,152,153]. The binding of the grape extract polyphenol myricetin to monomeric Aβ42 following the inhibition of amyloid aggregation was identified [152]. We investigated how myricetin altered the structural dynamics of Aβ42 amyloid fibril formation using HS-AFM. The observation was initiated by administering 2.5 µM Aβ42 (19:1 = LMW:seeds) and 10 µM myricetin to the HS-AFM sample chamber [93]. Some seeds appeared on the stage and they barely extended (Figure 11a) [93]. Then, the solution in the sample chamber was replaced with fresh 2.5 µM Aβ42 containing neither seed nor myricetin [93]. Immediately after the exchange, the fibrils started to grow, and the fibril amount also increased (Figure 11b) [93]. This result indicated that myricetin reversibly inhibited the fibril elongation reaction [93]. Further, the HS-AFM results suggested that the myricetin–monomer Aβ42 complex perturbed the dynamic equilibrium between the monomer and LMW that restricted the recruitment of LMW into the fibril ends. This could be by either the unavailability of seed competent Aβ42 species to recruit into the fibril ends or the reversible binding of myricetin to the fibril ends or both.

### 6.3. Aggregation Inhibition by Synthetic Polymers

Amylin (also known as islet amyloid polypeptide protein (IAPP)) is a 37-residue peptide that is produced and co-secreted with insulin from pancreatic β cells. The amylin amyloid aggregates deposit on the β cells in the type II diabetes. Amylin shares some common characteristics with Aβ, such as folding into similar β-sheet structures [154], binding to amylin-3 receptor [155], and being digested by insulin-degrading enzyme [156]. Amylin crosses the blood–brain barrier (BBB) [157,158,159], and its aggregate deposition is found in the brains of type II diabetes patients with AD [121]. The mechanisms underlying the pathological [122,123] and suppressive [117,118,119,120] effects of amylin to AD remain controversial [160].

**Figure 12:**
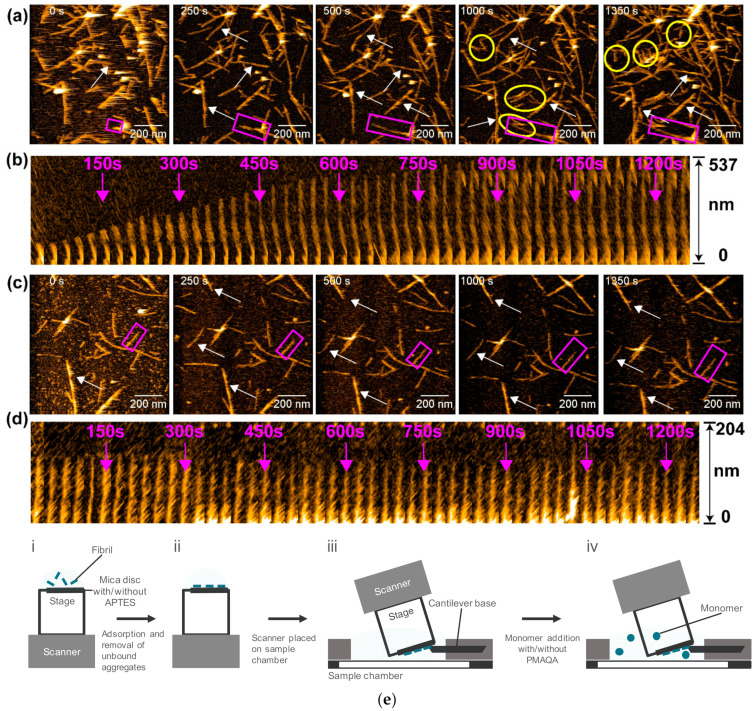
HS-AFM observation of amylin fibril growth. (**a**,**c**) HS-AFM images of the amylin seeding reaction in the absence (**a**) or presence (**c**) of polymethacrylate-derived copolymer (PMAQA). The de novo nucleated fibrils are highlighted by yellow circles in (**a**). The growth extents of fibrils are indicated by the white arrows. (**b**,**d**) Kymographs of individual amylin fibrils highlighted by purple boxes in (**a**) and (**c**) in the absence (**b**) or presence (**d**) of PMAQA. The experimental setups are shown in (**e**). Reprint with permission [116]: Copyright (2019), the Royal Society of Chemistry.

We observed amylin aggregation and fibril formation in the presence or absence of a polymethacrylate-derived copolymer (PMAQA) that has been used in various biological research fields, including lipid–nanodisc formation, Aβ–nanodisc interaction, the improvement of drug delivery, and the bioavailability on microencapsulation [41,161,162,163]. In this observation, amylin fibril seeds were immobilized on the stage beforehand, and then amylin monomers were added to the sample chamber alone or together with PMAQA [116]. In the absence of PMAQA, the original fibril seeds grew as observed in Aβ42, and some of the newly created fibrils in the chamber bound to and extended on the stage (Figure 12a,b) [116]. In the presence of PMAQA, the original fibril did not elongate, and de novo fibrils did not appear (Figure 12c,d) [116]. Unlike the HS-AFM observations for Aβ42 that showed fiber polymorphism (Figure 2), amylin showed a majority of straight fibers (Figure 12). NMR analysis of the PMAQA–amylin complex indicated that PMAQA bound to the amyloid core domain (NFGAIL) of amylin [116]. These results suggested that the binding of PMAQA to amylin monomer inhibited nucleation and self-replicative fibril elongation [116]. In this way, the consecutive imaging of fibril seeds adhered on the stage in advance and in the same observation area after the addition of monomers could not only observe fibril elongation immediately, but also distinguished between the original fibrils and the de novo fibrils by identifying the appearance time and place of the fibrils.

The effects of styrene–maleic acid copolymers varying with charge (SMAEA/SMAQA) on amylin were investigated using HS-AFM [164]. The results from this study identified morphologically distinct amylin species. HS-AFM showed the cationic SMAQA polymer [165] interaction with amylin generates de novo spherical globulomers that are incompetent to grow in size or recruit to the fibril ends to proceed with the seeding reaction. In contrast, the anionic SMAEA polymer [166] accelerated amylin fibrillation and generated de novo spherical globulomers that grew in size and proceeded with the seeding reaction. These observations indicate that SMAQA and SMAEA acted as an inhibitor and promotor for amylin aggregation, respectively.

### 6.4. Aggregation Inhibition by Heterologous Aggregation

Although the heterologous aggregation of amyloidogenic proteins has been found in vivo, the molecular mechanism has been still unclear. In addition, designing peptides that lead to coaggregation with amyloidogenic proteins can potentially become the candidates for the therapeutic drugs.

**Figure 13:**
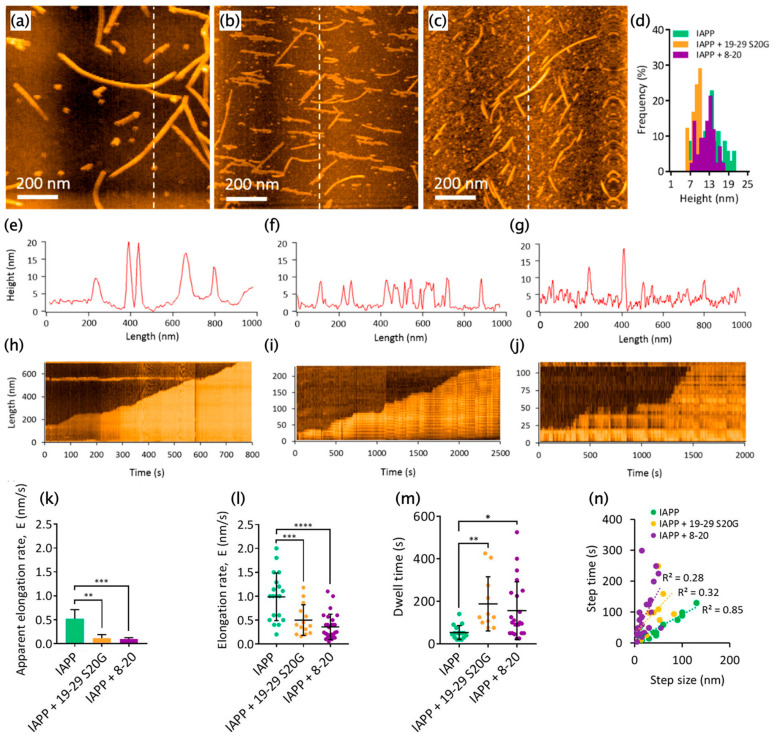
HS-AFM observation of homologous and heterologous amylin fibril growth. (**a**–**c**) HS-AFM images of self-assembly of full-length amylin (**a**), coaggregations with 19–29 S20G (**b**) and 8–20 (**c**). (**d**) Distribution of fibril thickness. (**e**–**g**) Cross-sections at the dashed lines in (**a**–**c**). (**h**–**j**) Representative kymographs of homologous full-length amylin fibrils (**h**), heterologous fibrils with full-length amylin/19–29 S20G (**i**), and with full-length amylin/8–20 (**j**). (**k**) Apparent fibril growth speed. (**l**) Pause-free elongation speed. (**m**) Distribution of pause time. (**n**) Relationship between the step size and step time. Error bars correspond to the mean ± S.D. Asterisks represent statistically significant differences between the sample mean and control mean (ANOVA; * *p* ≤ 0.05; ** *p* ≤ 0.01; *** *p* ≤ 0.005; **** *p* ≤ 0.001). Reprinted with permission from [108]. Copyright (2019) American Chemical Society.

Kakinen et al. observed the structural dynamics of homologous fibril growth of full-length amylin and the heterologous assembly of full-length amylin/its shorter component, 8–20 or 19–29 S20G, using HS-AFM [108]. The 8–20 and 19–29 S20G are the peptides from the 8th to the 20th and from the 19th to the 29th with replacement at the 20th residue of serine with glycine. Both of the two regions build up a cross β structure in amylin fibrils [108]. Using HS-AFM, they found and characterized the inhibition of amylin fibril growth in coaggregation with the peptides [108]. The fibril morphology of pure full-length amylin differed from that of coaggregation (Figure 13a–g) [108]. The fibril thickness of homologous full-length amylin was widely distributed from 9 to 20 nm, while that of the coaggregation fibrils showed a narrower distribution (Figure 13d) [108]. The authors interpreted that the mature fibrils formed at the pure full-length amylin on the HS-AFM stage, which was reflected in the wide distribution of fibril thickness (Figure 13d) [108].

Compared with the homologous aggregation, the coaggregation increased the surface roughness, which resulted from the higher production of small aggregates (Figure 13a–c,e–g). Similar to our Aβ42 study [93], kymographs showed that both the homologous and heterologous fibril growth of amylin followed the stepwise and polarized manner at the fast and slow ends (Figure 13h–j). The kymograph analysis also indicated that the coaggregation reduced the apparent growth speed (Figure 13k) due to decreases in the step speed (Figure 13l) and increases in the pause time (Figure 13m) [108]. These results were interpreted as the incorrect docking of the shorter peptides that should be removed or converted to the correct docking, which reduced the fibril elongation [108].

In addition, the authors focused on the relationship between step size and step time. This proportionality was much higher in the self-elongation of full-length amylin than in the coaggregation (Figure 13n) [108]. This analysis suggested that the step speed was kept at a constant value in the self-assembly of full-length amylin, while, for the coaggregation, the step speed was widely distributed [108]. They also showed significant recovery effects of coaggregation with 19–29 S20G on the survival rate, hatching rate, and phenotypic normality, using an in vivo model with zebrafish embryos [108].

## 7. HS-AFM Observation of Other Amyloidogenic Proteins

High-speed AFM reveals the structural dynamics of not only Aβ and amylin, but also other amyloid protein aggregations. Milhiet et al. showed protofilament elongtation and its stacking into polymorphic mature fibrils of lithostathine, which is overexpressed in the pre-clinical stage of AD [115]. Zhang et al. studied the structural dynamics of α-synuclein monomers and dimers using HS-AFM [110]. The monomer showed a transition between a spherical structure and a protruding tail-like structure and a structure completely extended, similar to a string [110]. The dimers were less flexible and basically maintained a dumbbell structure in which two spherical structures were connected [110]. Konno and Watanabe-Nakayama et al. observed yeast prion Sup35 monomer, oligomer, and fiber elongation [114]. HS-AFM revealed the structural dynamics of the intrinsically disordered (IDR) and partially folded regions of the Sup35 monomer, differences in the core structure and in the IDR between Sup35 oligomers and fibrils, the stepwise growth of oligomers with distinct core size, and the continuous unidirectional elongation of Sup35 fibrils [114].

## 8. Optimization of HS-AFM Observation of Amyloid Aggregation

HS-AFM observation depended on the sample preparation, surrounding buffer composition, and imaging parameters. These conditions should be optimized so that the structure and dynamics on the HS-AFM stage are consistent with the conventional structural and dynamic analyses. In this section, we discuss the conditions for HS-AFM observation of amyloid aggregation.

### 8.1. Sample Preparation and Control of Aggregation Initiation

The sample preparation procedure should be optimized according to the aggregation process to be observed by HS-AFM. As shown in Section 2 and Section 4, structural dynamics differ depending on the preparation of Aβ42 (LMW and HMW). In addition, the operational efficiency at the start of HS-AFM observation and the reproducibility of the results should be considered. The sample should be stored under conditions in which the aggregation does not progress, and the aggregation reaction needs to proceed during HS-AFM observation. In our studies, the size fractionated Aβ42 samples were stored in low ionic strength 10 mM sodium phosphate, at pH 7.4 [93,150], and then the aggregation was initiated by the addition of 0.1 M sodium chloride or potassium chloride [93].

### 8.2. Sample Density

Since the size of the HS-AFM observation field is limited as described in Section 8.4, a low density of sample molecules makes it difficult to find the molecules to be observed, while a high density means that the molecules are in contact with other molecules and have constrained structural dynamics; both cases should be avoided. For example, when the growing end of an observed fibril encounters another fibril, the growth stops there, and no further elongation occurs (Figure 1b). For the statistical analysis described in Section 2.1 and Section 4.2, a sufficient number of aggregates are required within the observation area. Considering these conditions, the optimization of sample concentration is important. In our study, we set the concentration of Aβ42 to 2.5 µM in terms of monomers as in [93].

### 8.3. Stage Materials and Sample Solution

HS-AFM can only visualize the molecules at the interface between the solid surface of the stage and the liquid phase of the sample solution. HS-AFM cannot image molecules that are weakly bound to the stage and that are free in the sample solution. In addition, conditions in which molecules bind strongly to the stage could affect the structural dynamics and thus should be avoided. Therefore, we optimized a sample molecule binding condition in which the molecules stay at their positions during the time required to acquire their images in one frame, and where the structural dynamics are not affected (or the effect on the structural dynamics can be estimated). The binding force to the stage was determined by the chemical properties of the sample molecules (isoelectric point (pI), hydrophobicity, etc.), stage material, and composition of the sample solution. Thus, depending on the properties of the target molecule under investigation, one can optimize the chemical property of the mica surface (e.g., bare mica or 3-aminopropyltriethoxysilane (APTES) modified mica) and/or solution pH and ionic strength to obtain the desirable structural and dynamic information.

An atomically flat surface of freshly cleaved mica ([KAl_2_(OH)_2_AlSi_3_O_10_]) was used as a first candidate for the HS-AFM stage. The surface was negatively charged. The pH and ionic strength of the solution and the pI of the sample molecules determined the electrostatic interaction with the mica surface. As the pI of Aβ42 was 5.31 (estimated by the ProtParam [167] in Expasy), the net charge was negative in a neutral pH solution, and electrostatic repulsion was expected to act between the peptide surface and mica. In fact, we observed Aβ42 to be less adsorbed to mica in a low ionic strength buffer of 10 mM sodium phosphate, pH 7.4.

Electrostatic interactions between mica and sample molecules can be controlled by the addition of salts, as observed in ion exchange chromatography. Aβ42 aggregates bound to the mica surface immediately after the addition of 0.1 M sodium chloride to its low ionic strength buffer solution (10 mM sodium phosphate, pH 7.4), suggesting that the electrostatic repulsion acting between the Aβ42 and mica surface was canceled by the addition of salt and the hydrophobic interaction bound Aβ42 to the mica surface. The effect of salt on the electrostatic interaction between sample molecules and the mica surface varied depending on the type of cation, even at the same concentration. Potassium ions had a greater canceling effect of this electrostatic interaction than did sodium ions [168,169]. We used this difference in potassium and sodium ions to change the interaction between Aβ42 and the mica surface to characterize the structure switch of Aβ42 fibril elongation, as described in Section 2.1.

Chemical modification of the mica surface or other materials can be used to modulate the interaction between sample molecules and the stage surface. Modification of the mica surface with 3-aminopropyltriethoxysilane (APTES) made the surface positively charged [170]. In addition, the amino group of APTES can be used for the covalent immobilization of sample molecules with glutaraldehyde [170]. Highly ordered pyrolytic graphite (HOPG) can be used for the immobilization of sample molecules with hydrophobic interactions. HOPG was used for the immobilization of Aβ25-35 fibrils [126].

**Figure 14:**
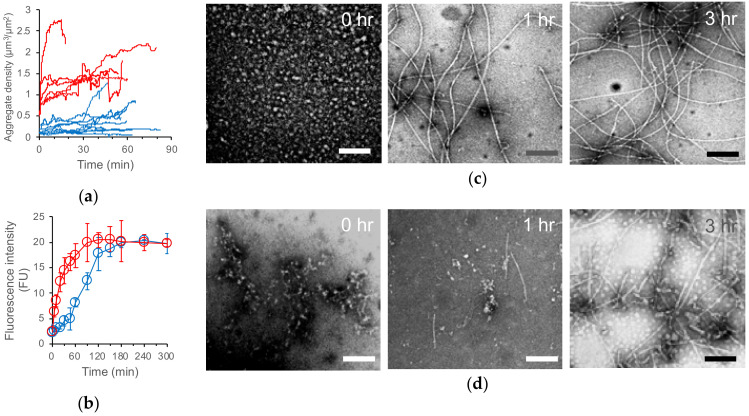
Relationship between HS-AFM observation, thioflavin T (ThT) assay, and transmission electron microscopy (TEM) of LMW and HMW Aβ42 incubation. The time evolution of the total Aβ42 aggregate density in HS-AFM observation (**a**); time course of ThT fluorescence intensity (b); TEM images of LMW (**c**; blue lines in (**a**,**b**)) and HMW ((**d**); red lines in (**a**,**b**)) Aβ42 incubations. Bars, 100 nm. Reproduced from [93].

The effect that the sample-stage interaction had on the structural dynamics of sample molecules needed to be verified. To examine this effect, we confirmed the consistency between the time courses of the ThT assay in the in vitro aggregation reaction and the total amount of aggregate in the HS-AFM observation area. The trends of the total aggregate amount and the ThT fluorescence intensity were consistent with each other (Figure 14a,b) [93]. They rapidly increased in the HMW Aβ42 incubation and gradually increased at a low level in the LMW Aβ42 incubation (Figure 14a,b) [94]. The aggregate structures were also consistent between the HS-AFM image and the transmission electron microscope (TEM) image observed at the same time points after the initiation of aggregation (Figure 1b, Figure 6a and Figure 14c,d). For our HS-AFM observation of the amylin aggregation, we used a low ionic strength solution and confirmed no significant difference in observation between bare and APTES mica, indicating that the electrostatic interaction between the sample and the surface did not affect the structural dynamics of amylin in our observation [116].

### 8.4. Size of Scanning Area and Time for Image Acquisition

The observation area and imaging speed are in an inversely proportional relationship. For the statistical analysis of structural dynamics of amyloid aggregates, the size of the observation area is needed prior to the imaging speed. In this case, the typical scale of the observation field and the typical imaging speed are micrometers and several seconds to ten seconds per frame, respectively. For the imaging of faster structural dynamics of individual aggregates, the typical imaging speed is subseconds per frame, which requires a reduction in the observation area. The time ‘T’, also referred as the scanning rate, for the acquisition of one frame in the observation area (W nm in width × N lines in the y-direction) is expressed as follows [170]:

T = πWN/(2λf_B_θ_m_) (3)

where λ is the periodicity of the sample surface with the sinusoidal shape, f_B_ is the feedback bandwidth, and θ_m_ is the maximum allowable phase delay of feedback control. λ is usually several nm, f_B_ is about 100 kHz at maximum, and θ_m_ is ≈20° or less for fragile samples and ≈45° or more for stiff samples. In addition to Equation (3), the number of frames captured in the same observation field should be considered. The tapping HS-AFM probe applies a mechanical force to the sample molecules. Thus, the larger the number of frames, the higher the probability that the sample will be broken. In addition to the imaging speed and applied force, the number of frames should be reduced for the analysis of increasing numbers of amyloid fibrils because the fragmented amyloid fibrils individually serve as seeds and then grow.

## 9. Conclusions

Different Aβ fibril structures have been found in different AD patients, which evokes the relationship between the fibril formation and AD progression. The structural dynamics of amyloid aggregation need to be elucidated for diagnosis and drug discovery. In previous studies, the structural dynamics were characterized by separately studying the structure and dynamics. However, the conventional structure and dynamics analyses lack information from the other missing component. Thus, the information that could not be gathered from those studies has remained unknown. The development of HS-AFM allowed video recording with nanometer spatial resolution, which has enabled the simultaneous analysis of structure and dynamics. We were able to analyze the structural dynamics of individual amyloid aggregates, including fibrils, even when different types of aggregates coexisted.

Recent studies have shown that amyloid proteins formed aggregates with diverse structures under physiological conditions that differed from those in vitro [171]. Under physiological conditions, amyloidogenic proteins underwent interactions with biological membranes, metal ions, other amyloidogenic proteins, and variants with different amino acid sequences causing heterologous aggregation. When reproducing the various physiological conditions by immobilizing a planar membrane on the stage and/or adding metal ions or variants of amyloidogenic proteins to the sample chamber, HS-AFM was used to visualize the structural dynamics in the aggregation processes. In addition, comparison with the observations in the presence of designed inhibitors may contribute to therapeutic development to characterize the desired drug targeting specific toxic aggregates.

HS-AFM observation has some unique characteristics: the visualization was limited to the structural dynamics that occurred in the solid–liquid interface of the stage and the sample solution; the imaging speed and size of the observation area were restricted by their inverse proportion relationship; and the statistical analysis and the macroscopic trend of the HS-AFM observations must be confirmed to be consistent with the conventional structure and dynamics analyses. In this way, HS-AFM did not only link the previous structure and dynamics analyses but did identify the structural dynamics that could not be elucidated using conventional methods.

## Abbreviations

AD	Alzheimer’s disease
Aβ	Amyloid β
HS-AFM	High-speed atomic force microscopy
HMW	High molecular weight
LMW	Low molecular weight
ThT	Thioflavin T
